# The effect of planned presence of the family at the time of weaning on the length of weaning from mechanical ventilation in patients with brain injury admitted to intensive care units

**DOI:** 10.1186/s12912-022-01098-4

**Published:** 2022-11-28

**Authors:** Fatemeh Salmani, Maryam Moghimian, Mina Jouzi

**Affiliations:** Nursing & Midwifery Sciences Development Research Center, Najafabad Branch, Islamic Azad University, Najafabad, Iran

**Keywords:** Weaning, Family, Intensive care unit (ICU), Brain injury, Mechanical ventilation

## Abstract

**Background:**

Weaning the patient from mechanical ventilation (MV) is one of the common treatments in intensive care units (ICU). Among the factors that can complicate the weaning process are psychological problems caused by a lack of family visits.

**Objective:**

This study aimed to evaluate the effect of scheduled visiting on weaning from mechanical ventilation in patients with brain injury admitted to ICUs.

**Methods:**

This quasi-experimental study was performed on 60 patients with brain injury referred to one of the hospitals in Central Province, Iran in 2020. The selection of samples based on inclusion criteria and random allocation to the two groups of intervention and control by permuted block randomization was done. The first-degree relatives of the patients at the time of weaning in the intervention group were present at the patient’s bedside twice a day at 10:00 AM and 3:00 PM for 30–45 min and performed what they had been trained to do. Participants in the control group visited patients from the area outside the patient room. Length of weaning, length of mechanical ventilation, and length of stay in the ICU were recorded and measured using a researcher-conducted checklist. The results were analyzed using descriptive and inferential statistics and chi-square and independent t-tests in SPSS16.

**Results:**

The study results showed that the number of patients weaned from mechanical ventilation in the intervention group was significantly higher than that of the control group (*P* ≤ 0.05). The mean length of weaning in the intervention and control groups was not statistically significant, but it was clinically significant in the intervention group (*P* > 0.05). In addition, the length of mechanical ventilation and the length of stay of the intervention group in the ICU were significantly shorter than that of the control group (*P* ≤ 0.05).

**Conclusion:**

According to the study results, scheduled visiting resulted in faster weaning from mechanical ventilation. As a result, nurse managers are suggested to make arrangements for family members to be present at the patient’s bedside in order for patients to be weaned off the mechanical ventilator more easily.

## Introduction

Weaning from mechanical ventilation (MV) is one of the important stages of treatment in intensive care units (ICUs). Weaning process accounts for about 40% of the length of MV. Weaning is the process of reducing ventilator support and ultimately resulting in a patient breathing spontaneously and being extubated [1]. Numerous factors are involved in weaning from MV, including nutrition, proper communication with the patient, sedatives and narcotics, psychological factors, metabolic factors, weaning methods and measurable parameters [2]. Weaning from MV depends on the mental and physical condition of the person. Additionally, nursing interventions and environmental conditions can induce anxiety in patients. Anxiety in the patient increases the number of breaths, decreases the tidal volume and minute volume and the need for more sedatives in the patient [1]. Excessive use of sedatives can make the patient dependent on MV [2]. The presence of the family at the patient’s bedside can create a trusting and intimate relationship between the family and the patient. These can improve the parameters of the MV and reduce the need to use sedatives. For this reason, the need for a family in (ICUs) is more felt [3].

Family members can help the patient wean off the mechanical ventilator. Most of the mechanically ventilated patients experience a lot of anxiety and stress because they are treated aggressively, do not have many visitors, and are in a loud and strange place, which can make them reliant on MV. For those admitted to ICUs, sensory deprivation caused by the absence of family visits can lead to a greater reliance on MV [4]. Happ et al. [5] conducted a qualitative study and showed that families, who were present at the patient’s bedside during the weaning process, communicated with them through physical contact, touching, kissing and even supervision. The length of weaning (LOW) from the MV was significantly increased. They found that having a family was relaxing and useful during weaning from MV. Safaeepour et al. [4] conducted a study and showed longer weaning in patients whose family members were present at their bedside immediately after their admission to the cardiac ICU. Also, Burns et al. [6] showed that early family involvement could have many benefits for the patient’s weaning and prevent the complications of MV.

In other studies, patients considered the presence of family members effective in meeting their needs and feeling secure [7, 8]. Scheduled visiting has been shown to provide psychological support for patients, but Iranian hospitals have strict rules that put patients and their families under a lot of pressure [9]. Such limitations can slow down a patient’s recovery process, increase the length of MV, and have devastating psychological effects on both the patient and their family [10]. Weaning from MV is one of the most important tasks in ICUs, and the patient’s mental state can either accelerate or impede the weaning process, incurring enormous costs. Therefore, the present study aimed to determine the effect of scheduled visiting on the weaning from MV in patients with brain injury admitted to ICUs in order to provide a useful procedure for family involvement in patient care, improve health, and reduce healthcare costs.

## Method

### Study design

This quasi-experimental study was performed on two groups of intervention and control. The ICUs (trauma) of the public sub-specialty hospitals in Central Province, Iran, were selected for this study. The families can visit their patients three times a week (Monday-Wednesday-Friday) from an area outside the patient room, avoiding them to have close physical and emotional contact with patients.

### Participants

Mechanically ventilated patients aged 18–65 years, who had a brain injury with a level of consciousness between 5 and 8 were eligible. First-degree relatives (father, mother, sister, brother, spouse, and child) aged 18–65 years, who could communicate verbally and were willing to participate in the study met the inclusion criteria for families. Patients who died, were transferred to the ward, or had to undergo emergency surgery were all excluded. The sample size was 30 individuals in each group based on the results of Abbasi and Mohammadi [11], with 95% confidence and 80% test power, S1 = 0.9, S2 = 0.9, X1 = 6.8, and X2 = 7.8 (Consciousness level variable). By taking into account a ten-percent dropout, 33 subjects in each group were estimated. The selection of samples based on inclusion criteria and random allocation to the two groups done. Then, participants were assigned into the intervention and control groups by the permuted block randomization method. This random method involves a sequence of blocks, each block containing a number of predefined numbers or letters. Permuted block randomization method is a way to randomly assign a participant to a treatment group. While maintaining a balance between treatment groups. Each “block” has a certain number of random treatment instructions. The treatment groups in this study included (scheduled visiting) intervention group (number 1) and (routine ward appointments) control group (number 0). Six blocks of 10 were selected and according to the order of the numbers in these blocks, individuals were assigned to the intervention and control groups. The sampling and intervention process lasted from January 2021 to July 2022.

### Ethical considerations

All the families of the study participants were informed of the study objectives and provided informed written consent before participation. Participants’ families were free to enter or leave the study. They were also assured that their patient information would be kept confidential.

### Instruments

In order to collect data in this study, a researcher-conducted checklist of weaning from MV was used. The checklist includes: a) demographic information (age, sex, education and history of underlying diseases - use of narcotics and sedatives, dose of drugs, MV mode, type of brain trauma, cause of brain trauma) and b) indicators of weaning from MV (weaning mode, length of weaning (hours), length of MV (days) and length of stay in the ICU (days), which were measured during 7 days.

The content and face validity of the checklist was assessed by three anesthesiologists and seven ICU nurses. Inter-rater reliability of 0.83 was obtained.

### Intervention

When selecting a patient’s first-degree relative for the intervention (mother, father, spouse, or sibling), the family member with the strongest emotional ties to the patient was chosen. Two ICU nurses received the necessary training in family interventions, ward rules, and the importance of scheduled visiting. Then, they trained close family members about rules of ICU, patient’s condition, the value of scheduled visiting, hand washing before entering and after leaving the ward, talking to patient, and getting the patient to cough, breathe deeply and do breathing exercises (Table 1). The family member was present at the patient’s bedside twice a day at 11 am and 3 pm. The visiting lasted for 30–45 min for 7 days [11] from the beginning of admission (14 times in total). On all occasions, the patient’s assigned nurse accompanied and assisted the first-degree relative, making sure everything went as planned and providing assistance as needed. As soon as the family member’s mental and emotional condition became stable, a trained nurse helped them to implement the care plan.

**Table 1:** How to implement family care while being at the patient’s bedside

Selection of a close family member based on the closest proximity to the patient in consultation with the family	
Training of two nurses to train the family to attend the patient’s bedside	
Preparing the family to enter the department (familiarization with the department - familiarization with equipment and devices, etc.) and familiarization with the rules of the ICUs	
Family training for intervention by trained nurses (2-hour session) 1- Teaching the time of entering and leaving the ward and the presence of the family on time 2- Being a close family member for 7 days 3- Washing hands upon entering the ward and before doing anything for the patient (medical hand washing training) 4- How to communicate with the patient during 30 to 45 minutes - Greeting the patient and calling the patient by her name - Introducing yourself to the patient to be repeated several times - Making close contact with the patient, such as touching the face and skin contact with the patient and kissing the patient - Talking about the condition of other family members - Talking about the patient’s condition and what devices are connected to the patient. - Sit the patient down with the permission of the relevant nurse - Encourage the patient to breathe slowly and deeply - If the patient is conscious, encourage the patient to cough and swallow saliva - Encouraging the patient to hold his breath and let it out slowly in case of spontaneous breathing with the device - During the weaning of the patient from the device, continuous family communication with the patient and encouragement to breathe deeply and cough and expectorate - Sit the patient down and help the nurse for the patient’s respiratory physiotherapy - Making the patient perform incentive spirometry with the device 5- After finishing 45 minutes, kiss the patient goodbye and tell the patient about the time of the next appointment	

During their visit with the patient, the family member told the patient, who they were, as well as the date and location of the visit. Furthermore, the family member spoke to the patient in a soothing tone and had physical and emotional contact with the patient. After receiving permission from a nurse, the family member sat the patient down in a semi-sitting position, getting them to breathe slowly and deeply. They were also at the patient’s bedside when the patient was weaned off the ventilator. The family member told them to take deep breaths and cough.

Weaning of the patient from the MV is the responsibility of the doctor based on the mode of ventilation and the parameters of the MV, which are different for each patient based on the condition of the patients. The weaning process starts by reducing the parameters of the MV (number of breaths, fio2, and pressure support) and being on the SIMV mode, and if the patient tolerates the reduction of the MV parameters, then the patient mode is changed to the spontaneous mode. After that, if the spontaneous mode is tolerated, the patient is weaned from the MV and placed on the venturi tipis with sufficient oxygen. The patient is alternately placed on the machine and then placed on the venturi tipis. Gradually, the times of placement on the Venturi tipis increased so that the patient is completely placed on the venturi tipis within 24 h. If the patient tolerates the venturi tipis completely within 24 h, the patient’s tracheal tube will be removed and the patient will breathe with an oxygen mask and will be assessed within 24 h. In case of difficulty in breathing, the patient should be placed on the mechanical ventilator again.

### Control group

There was no scheduled visiting for the control group. Family members in the control group could visit their patients for 3–5 min. Sometimes, they had to visit their patients from an area outside the patient room or through closed-circuit television. After 7 days, the length of weaning (hours), the length of MV, and length of stay in the ICU (day) were compared in two groups.

### Data collection

Data were collected using a researcher-conducted checklist of weaning from MV twice a day for 7 days.

### Data analysis

The data were analyzed using SPSS16. Alpha significance level was considered 0.05. Demographic information of the research samples (nominal variables) was analyzed using Chi-square test, while quantitative variables were analyzed using independent t-test.

## Results

In this study, two patients in the intervention group were excluded from the study due to death and one patient due to transfer to other departments. In the control group, one patient died and two patients were excluded from the study due to emergency surgery. Finally, 30 patients remained in the intervention and control groups.

The chi-square test revealed that the distribution of samples in the intervention and control groups was the same in terms of sex, level of education, diagnosis, cause of admission, use of sedatives, and MV mode. In addition, the independent t-test showed no statistically significant difference in mean age between the intervention and control groups (Table 2).

**Table 2:** Comparison of demographic characteristics of study samples in intervention and control groups

**Demographics**	**Case**		**Control**		***p*-value**
	N	%	N	%	
**Sex**					
Men	22	73.3%	20	66.6%	**χ**^**2**^ **= .32** ***P*** **= .57**
Woman	8	26.7%	10	33.4%	
**Education**					
Under graduate	4	13.3%	4	13.3%	**χ**^**2**^ **= 2.08** ***P*** **= .55**
Diploma	18	60%	17	56.7%	
post graduate	8	26%	9	30%	
**Diagnosis**					
Cerebral Hemorrhage	16	53.4%	18	60%	**χ**^**2**^ **= 4.43** ***P*** **= .61**
MIX	4	13.3%	2	6.7%	
Contusion	6	20%	9	30%	
DAI: (Diffuse Axonal Injury)	4	13.3%	1	3.3%	
**Cause**					
Car accident	10	33.3%	11	36.7%	**χ**^**2**^ **= .74** ***P*** **= .68**
Motor accident	16	35.3%	17	56.7%	
Falling	4	13.3%	2	6.7%	
**Use of sedatives**					
Yes	28	93.3	27	90	**χ**^**2**^ **= .22** ***P*** **= .62**
No	2	6.7	3	10	
**Modes**					
SIMV	21	70	20	66.6	**χ**^**2**^ **= 3.39** ***P*** **= .33**
SPONT	8	26.6	5	16.7	
PSV	1	3.4	5	16.7	
	**Mean (SD)**		**Mean (SD)**		**t = .18** ***P*** **= .85**
Age	35.4(14.2)		32.9(13.6)		

The chi-square test showed that the frequency distribution of weaning from MV was significantly different between intervention and control groups (*P*-value = 0.05), so that weaning from the MV in the intervention group was significantly longer than that of the control group (Table 3).

**Table 3:** Comparing the weaning from mechanical ventilation in intervention and control groups during 7 days

Weaning	Case		Control		***p***-value
	N	%	N	%	
**Yes**	13	43.3	6	20	χ^2^ = 3.77 *P* = .05
**No**	17	56.7	24	80	

Independent t-test showed that the mean length of weaning (hours) was not significantly different between the intervention and control groups (*P*-value = 0.26), while this difference was clinically significant in the intervention group. Independent t-test showed that the mean length of MV in the control group was significantly longer than that of the intervention group (*P*-value = 0.03). In addition, independent t-test showed that the mean length of stay in the ICU was significantly different between the intervention and control groups (*P*-value = 0.03). Patients in the intervention group spent less time in the ICU than those in the control group (Table 4).

**Table 4:** Comparing the mean length of weaning (hours), length of mechanical ventilation (days) and the length of stay in the ICU in the intervention and control groups

Variebles	Case		Control		***p***-value
	Mean	SD	Mean	SD	
Length of Weaning/hours	2.1	2.7	1.3	2.5	**t = 1.33** *P* = .26
Length of Mechanical ventilation /Day	6	4.2	10.3	7.3	**t = 2.2** *P* = .03
Length of stay ICU/Day	15.1	6.1	22.3	7.2	**t = 2.14** *P* = .03

The results of two-way analysis of variance showed that gender and diagnosis did not have a statistically significant effect on the length of weaning. Also, the factor of gender and diagnosis had not a statistically significant effect on the length of stay in the ICU (Table 5).

**Table 5:** Determining the factor of sex and diagnosis on length of weaning (hours) and the length of stay in the ICU

**Sex**	**Diagnose**	length of weaning		***p*-value**
		**Mean**	**SD**	
Men	Cerebral Hemorrhage	1.43	2.1	Sex & length of weaning F = .17 *P* = .68
	MIX	0	0	
	Contusion	2.1	3.1	
	DAI	0	0	
Woman	Cerebral Hemorrhage	1.5	1.32	
	MIX	0	0	Diagnose & length of weaning F = 1.6 *P* = .15
	Contusion	4.2	3.3	
	DAI	0	0	Sex & Diagnose & length of weaning F = .25 *P* = .93
**Sex**	**Diagnose**	length of stay in ICU		***p*-value**
		**Mean**	**SD**	
Men	Cerebral Hemorrhage	19.8	5.9	Sex & length of stay in ICU F = .08 *P* = .77
	MIX	23.80	10.18	
	Contusion	16.44	7.14	
	DAI	30.75	9.94	Diagnose & length of stay in ICU F = 2.7 *P* = .02
Woman	Cerebral Hemorrhage	21	6.1	
	MIX	30	4.2	
	Contusion	17	6.3	Sex & Diagnose & length of stay in ICU F = .99 *P* = .43
	DAI	25	9.9	

## Discussion

This study aimed to determine the effect of scheduled visiting on weaning from MV in patients with brain injury admitted to the ICU. The study results showed no statistically significant difference in all demographic characteristics between the intervention and control groups, indicating their ineffectiveness in the length of weaning and the length of MV in the intervention and control groups. The two groups were homogenized in terms of age, sex, type of brain injury, and the dose of sedatives because they could affect the length of weaning and MV [12–14].

The study results showed a significantly different frequency distribution in weaning from mechanical ventilator between the intervention and control groups, indicating that weaning from MV was significantly greater in the intervention group than in the control group. Furthermore, the study results showed that the mean length of weaning (hours) was not significantly different between the intervention and control groups, while this difference was clinically significant in the intervention group. Weaning of patients in the intervention and control groups was 2.1 and 1.3 h, respectively.

Perhaps the reason for the lack of statistical significance in the study is the presence of the family with the patient for a very short time, and if the presence of the family is continuously with the patient, the weaning time of the patient will increase.

Safaeepour et al. [4] studied the effect of family-centered care on weaning from MV in patients undergoing coronary artery bypass surgery. They showed longer weaning in patients whose family members were present at their bedside immediately after their admission to the cardiac ICU. In addition, the length of MV in the intervention group was shorter than that of the control group, indicating the importance of early weaning in all patients admitted to ICUs. Happ et al. [5] showed that family presence during weaning process led to satisfactory results in patients and their families. Burns et al. [6] also indicated that family involvement in weaning process was a new research model in ICUs. They showed that early family involvement could have many benefits for the patient’s weaning and prevent the complications of MV. The mentioned studies supported the present study, showing the effect of family involvement and scheduled visiting on the weaning process.

However, other studies showed that the use of patient-centered method (nursing care) was more effective in patients under prolonged MV. The reason for this discrepancy is the type of patients, who were under prolonged MV, which can complicate the weaning process. They concluded that barriers to family-centered care included lack of teamwork and resources [15].

The study results also showed that the mean length of MV (days) in the control group was significantly longer than that of the intervention group. In addition, the frequency distribution of the length of MV in the intervention and control groups showed that most of the patients in the intervention group were weaned off under 72 h, while most of the patients in the control group were weaned off for more than 7 to 10 days. Eriksson and Bergbom [16] found that the length of MV in patients who had visitors was longer than in those who did not. This discrepancy may be due to the type of patients admitted to general ICUs. Scheduled visiting was done with patient’s admission to ICUs, which reduced the length of MV in the patients of the present study. Khalafi et al. [17] showed that communication with the patient was very important during the weaning process. They found the category of family involvement in the care process. In this study, nurses asked family members to be present at patients’ beds and provide care for patients. In the present study, nurses asked influential family members to motivate patients, raise their moods, have regular verbal and tactile communication with them, and talk about pleasant daily events. To encourage weaning from MV, nurses closely monitored family members and allowed them to spend time with patients in case they did not have an adverse impact on the care process. Khalafi also believed that identifying the patient’s motivations for weaning, personal preferences, and coping styles, as well as choosing an appropriate strategy for easy weaning were important factors, which were realized with the family presence at the patient’s bedside [17]. Schou and Egerod [3] found that continuous communication with the patient facilitated the weaning from MV. Conversely, poor communication made the patient anxious and led to slow recovery and prolonged length of MV. Soleimani et al. [18] showed that communication and sensory stimulation by the patient’s family improved the patient’s respiratory status and ventilator parameters, but it had no effect on the weaning process. The reason for this discrepancy was family visiting once a day as well as the type of patients admitted to the ICU.

According to various studies, visiting in ICUs allows the family to take a greater share of patient care and leads to more opinions and a better understanding of the patient’s condition [19, 20]. As a result, it reduces family anxiety and increases their ability to support the patient and participate in decision-making about patient care [21]. Some countries’ visiting policies include respect for the individual, adherence to the principles of autonomy, kindness, and non-harm to the patient. According to current knowledge, the presence of family members cannot be a threat to the patient, rather it has a beneficial effect on the patient and family. From a moral point of view, limited visiting is not justifiable. The only exception is when the visitor may compromise the patient [22]. Various studies found the effect of family on the level of consciousness and faster recovery of the patients with brain injury [23, 24].

The study results showed that the mean length of stay in the ICU was significantly different between the intervention and control groups. The patients in the intervention group spent much less time in the ICU than those in the control group.

Eriksson and Bergbom [16] examined effect of visiting on patient outcomes in ICUs and found that length of stay in ICUs was not significantly different between patients who had visitors and those who did not. Patients who had visitors, on the other hand, had a significantly higher survival rate than those without visitors. Junior et al. [20] conducted a meta-analysis on 16 studies and showed that flexible family visit had no effect on the length of stay in the ICU. The type of ICU and the patients admitted to these wards are the reasons for the discrepancy between this study and the current one. Patients with brain injuries begin regenerating their brains as soon as they are admitted to the hospital, and this process is influenced by sensory stimulation. From the time of admission, visitors can have an impact on a patient’s recovery. Rezaei et al. [25] found no difference in the length of stay in the ICU between the intervention group, who had scheduled visiting once a day in the evening and the control group. The number of visitors to the ICU and the type of diagnosis of the patients included in the study may explain the disparity between our study and the mentioned studies.

Due to the outbreak of COVID-19 and the difficulty of entering the ICU, researchers had to use multiple hospitals for sampling in this study. In addition, the weaning process can be affected by individual differences in acute physiological severity, which is out of the researcher’s control.

## Conclusion

The study results showed that scheduled visiting could make it easier for patients to stop using MV and speed up their recovery when their families were with them, which was shown by the shorter length of stay in the ICU. Therefore, unrestricted visiting is suggested to help patients get better care with the help of their families. Also, considering that there is little research on family presence in ICUs during patient weaning, it is suggested that more research be done in this field.

## Data Availability

The data are available upon request to the corresponding author after signing appropriate documents in line with ethical application and the decision of the Ethics Committee.

## References

[1] Baptistella AR, Sarmento FJ, da Silva KR, Baptistella SF, Taglietti M, Zuquello RA, Filho N (2018). Predictive factors of weaning from mechanical ventilation and extubation outcome: a systematic review. J Crit Care.

[2] Ioannou GN, Locke E, Green P, Berry K, O’Hare AM, Shah JA, Crothers K, Eastment MC, Dominitz JA, Fan VS (2020). Risk factors for hospitalization, mechanical ventilation, or death among 10 131 US veterans with SARS-CoV-2 infection. JAMA Netw Open.

[3] Schou L, Egerod I (2008). A qualitative study into the lived experience of post-CABG patients during mechanical ventilator weaning. Intensive Crit Care Nurs.

[4] Safaeepour L, Mokhtari Nouri J, Moradian ST, Saied Ghiasi SM (2017). The effect of family-centered care on the duration of weaning from mechanical ventilation in coronary artery bypass surgery patients: a clinical trial study. Crit Care Nurs J.

[5] Happ MB, Swigart VA, Tate JA, Arnold RM, Sereika SM, Hoffman LA (2007). Family presence and surveillance during weaning from prolonged mechanical ventilation. Heart Lung.

[6] Burns KE, Devlin JW, Hill NS (2017). Patient and family engagement in designing and implementing a weaning trial: a novel research paradigm in critical care. Chest..

[7] Jacobowski NL, Girard TD, Mulder JA, Ely EW (2010). Communication in critical care: family rounds in the intensive care unit. Am J Crit Care.

[8] Boerenbeker P, Brandén AS, Chaboyer W, Hilli Y, Johansson L. Family member's experiences with and evaluation of an ICU liaison nurse service: a qualitative study. Nurs Crit Care. 2022. 10.1111/nicc.12775 35396916

[9] Haghbin S, Tayebi Z, Abbasian A, Haghbin H (2011). Visiting hour policies in intensive care units, southern Iran. Iran Red Crescent Med J.

[10] Bélanger L, Bussières S, Rainville F, Coulombe M, Desmartis M (2017). Hospital visiting policies-impacts on patients, families and staff: a review of the literature to inform decision making. J Hosp Admin.

[11] Abbasi M, Mohammadi E, Sheaykh RA (2009). Effect of a regular family visiting program as an affective, auditory, and tactile stimulation on the consciousness level of comatose patients with a head injury. Jpn J Nurs Sci.

[12] Burns SM, Fisher C, Tribble SS, Lewis R, Merrel P, Conaway MR, Bleck TP (2010). Multifactor clinical score and outcome of mechanical ventilation weaning trials: Burns wean assessment program. Am J Crit Care.

[13] Fonseca L, Vieira FN, Azzolin KD (2014). Factors associated to the length of time on mechanical ventilation in the postoperative period of cardiac surgery. Rev Gaucha Enferm.

[14] Jalalian HR, Aslani J, Panahi Z. Factors affecting the duration of mechanical ventilation device isolation of patients in intensive care units. Trauma Mon. 2009;14(3):163–8.

[15] Cederwall CJ, Olausson S, Rose L, Naredi S, Ringdal M (2018). Person-centred care during prolonged weaning from mechanical ventilation, nurses’ views: an interview study. Intensive Crit Care Nurs.

[16] Eriksson T, Bergbom I (2007). Visits to intensive care unit patients–frequency, duration and impact on outcome. Nurs Crit Care.

[17] Khalafi A, Adarvishi S, Soltani F (2020). Communication with chronic patients weaning from mechanical ventilation: a qualitative study on Iranian ICU caregivers. Prev Care Nurs Midwifery J.

[18] Soleimani M, Heidari S, Ghorbani R, Malek F (2017). Effect of sensory stimulation on respiratory function of patients undergoing mechanical ventilation. Koomesh..

[19] Manici M, Ghillani F (2018). Visiting policies in ICUs. Nursing in critical care setting.

[20] Junior AP, Besen BA, Robinson CC, Falavigna M, Teixeira C, Rosa RG (2018). Flexible versus restrictive visiting policies in ICUs: a systematic review and meta-analysis. Crit Care Med.

[21] Garrouste-Orgeas M, Philippart F, Timsit JF, Diaw F, Willems V, Tabah A, Bretteville G, Verdavainne A, Misset B, Carlet J (2008). Perceptions of a 24-hour visiting policy in the intensive care unit. Crit Care Med.

[22] Giannini A (2010). The “open” ICU:not just a question of time. Minerva Anestesiol.

[23] Kalani Z, Pourkermanian P, Alimohammadi N (2016). The effect of family guided visits on the level of consciousness in traumatic brain injury. J Biol Todays World.

[24] Moattari M, Shirazi FA, Sharifi N, Zareh N (2016). Effects of a sensory stimulation by nurses and families on level of cognitive function, and basic cognitive sensory recovery of comatose patients with severe traumatic brain injury: a randomized control trial. Trauma Monthly.

[25] Rezaie H, Sadeghi T, Abdoli F (2016). The effects of scheduled visitation on the physiological indices of conscious patients admitted at intensive care units. Evid Based Care.

